# Random resampling numerical simulations applied to a SEIR compartmental model

**DOI:** 10.1140/epjp/s13360-021-02003-9

**Published:** 2021-10-25

**Authors:** Francisco Morillas, José Valero

**Affiliations:** 1 Departament d’Economia Aplicada, Facultat d’Economia, Universitat de València, Campus dels Tarongers s/n, 46022 València, Spain; 2 Centro de Investigación Operativa, Universidad Miguel Hernández de Elche, Avda. Universidad s/n, 03202 Elche, Alicante Spain

## Abstract

In this paper, we apply resampling techniques to a modified compartmental SEIR model which takes into account the existence of undetected infected people in an epidemic. In particular, we implement numerical simulations for the evolution of the first wave of the COVID-19 pandemic in Spain in 2020. We show, by using suitable measures of goodness, that the point estimates obtained by the bootstrap samples improve the ones of the original data. For example, the relative error of detected currently infected people is equal to 0.061 for the initial estimates, while it is reduced to 0.0538 for the mean over all bootstrap estimated series.

## Introduction

In practice, when we analyze a sample of observed values of some variables, we need to take into account that such data are usually subject to errors of random character. Thus, there is a certain amount of uncertainty we have to deal with.

Resampling is a technique that allows us to estimate the uncertainty of a sample without making assumptions about it like the distribution type, sample size, etc. Basically, a bootstrap sample is a replicate sample of the original one of the same size, which is made using replacement. As a result, we obtain multiple replicates of the original data. Resampling has been applied in many fields such as statistics, operations research, mathematical programming, deep learning, biology, engineering and social sciences among others, as shown by the quantity of papers published (65,422 papers from 2015 or 128,512 from 1998, indexed only in Science Direct, as a result of searching for the keyword “bootstrap”). Despite the large number of articles published on this topic, to our knowledge, bootstrap has not been applied to SEIR models for the treatment of uncertainty. The classical references in this area are the works of Efron [1] and Efron and Tibshirani [2]. Recent applications in other areas can be found, for example, in [3–15].

Our aim in this paper is to apply resampling to a compartmental model of differential equations in order to improve the estimates of the variables in the presence of uncertainty. Compartmental models are ubiquitous in the modeling of processes in physics, biology and many other related sciences (see, e.g., [16, 17]). One of the first compartmental models in physics was used for the description of radioactive decay. Such models are very important in epidemiology, where the modelization of the spreading of viruses has given rise to the SIR models and its variants [17].

We apply resampling to a modified SEIR model, which was proposed in [18], in order to take into account the existence of an important number of undetected infected people in an epidemic (see [19] for another model of a similar type). In particular, we implement numerical simulations for the evolution of the COVID-19 epidemic in Spain from February 20, 2020, until May 17, 2020.

For our problem, we can take into account uncertainties of different nature. Some of them, as for example the changes in the speed of propagation due to environmental causes (the temperature, the humidity or the density of the population) or the individual strength to fight against the virus, are of random type. There are causes of uncertainty that are also random but are a consequence of human intervention such as, for instance, the confinement measures, the errors in the collection of information (concerning both the results of the PCR tests and the determination of the cause of death) or the impact on the survival rate of the progressive saturation of the health system. In this sense, in this work we try to increment the amount of information related to the data variability by means of resampling, as we are not able to measure the impact of the aforementioned random causes of uncertainty in the model. There is another methodology, which is appropriate only if we can assume that the observed data has no significant errors and the random fluctuations are due to the nature of the phenomenon under study. However, this situation is not the subject of the present paper and will be considered in a future work.

This paper is organized as follows.

In Sect. 2, we describe the system of differential equations which is used to estimate the evolution of an epidemic.

In Sect. 3, we implement first the methodology for estimating the parameters and the variables of the model, obtaining the particular numerical simulations for the COVID-19 spreading in Spain during the first wave of the pandemic in 2020. Second, we analyze the procedure which leads to the calculation of several bootstrap samples, showing the results for our model. Third, we define several measures of goodness which allow us to compare different estimations.

In Sect. 4, we show, by using the measures of goodness, that the point estimates obtained by the bootstrap samples improve the ones of the original data.

## Preliminaries: the model

SEIR models are classical compartmental models and are used widely in epidemiology [17]. We consider a modified SEIR model which was proposed in [18]. We distinguish between detected and undetected infected individuals, which leads to the following system of equations:

1 $$\begin{aligned} \dfrac{dS}{dt}&=-\dfrac{\beta \left( t\right) }{N}S\left( t\right) (1-\rho \left( t\right) )I\left( t\right) ,\nonumber \\ \dfrac{dE}{dt}&=\dfrac{\beta \left( t\right) }{N}S\left( t\right) (1-\rho \left( t\right) )I\left( t\right) -\sigma E\left( t\right) ,\nonumber \\ \dfrac{dI}{dt}&=\sigma E(t)-\left( \gamma _{1}(t)+\gamma _{2}(t)\right) I\left( t\right) ,\nonumber \\ \frac{dF}{dt}&=\gamma _{1}(t)\rho \left( t\right) I(t),\nonumber \\ \frac{dR}{dt}&=\gamma _{2}\left( t\right) \rho \left( t\right) I(t),\nonumber \\ \frac{dL}{dt}&=\left( \gamma _{1}(t)+\gamma _{2}(t)\right) (1-\rho \left( t\right) )I(t), \end{aligned}$$

where *S* is the number of susceptible individuals, *E* is the number of exposed individuals (it is assumed that they do not infect other people), *I* is the current number of infected people, *F* (*R*) is the number of dead (recovered) people among those who are detected and, finally, *L* is the number of both dead and recovered people among those who are not detected. We point out that only a fraction of infected people are detected, so the current number of infected individuals who are detected is given by:

2 $$\begin{aligned} D(t)=\rho \left( t\right) I\left( t\right) . \end{aligned}$$

Thus, $\rho \left( t\right)$ is the rate of detection of infected individuals at a given time. In this setting, assuming the ideal situation in which the detected individuals are put in quarantine and thus they are no able to infect other people, the number of individuals who are able to spread the disease is $I\left( t\right) -D\left( t\right) =(1-\rho \left( t\right) )I\left( t\right)$. This is why in the first two equations the variable $S\left( t\right)$ is multiplied by $(1-\rho \left( t\right) )I\left( t\right)$ and not by $I\left( t\right)$ as in the classical model.

Another important point is the fact that the coefficients of the model depend on time, which allows us to take into account that the rate of transmission is not constant, and moreover, it can change abruptly during an epidemic due to the restrictions imposed by the governments at different moments of time. We recall briefly the meaning of the coefficients. *N* is the size of the population (which is assumed to be constant), $\beta \left( t\right)$ is the average number of contacts per person per time, $1/\sigma$ is the average time of incubation of the disease (this coefficient is supposed to be constant), $\gamma _{1}\left( t\right)$ is the rate of mortality of detected people at moment *t*, whereas $\gamma _{2}\left( t\right)$ stands for the rate of recovery of detected people at moment *t*. We observe that $\gamma \left( t\right) =\gamma _{1}\left( t\right) +\gamma _{2}\left( t\right)$ is the rate of removal of infected individuals (because either they die or they recover) and that for simplicity this rate is supposed to be the same among the detected and undetected individuals. Also, $1/\gamma \left( t\right)$ means the average time after which an infected individual is removed.

## Methodology

This section is split into two main parts. In the first part, we introduce the method for estimating the parameters of problem (1) from a deterministic point of view. With this aim in mind, we determine the form of the functions defining the parameters of the model to be estimated and give the value of certain parameters on the base of previous studies. Also, we describe the heuristic algorithm called differential evolution, which will be used to obtain the values for the parameters that minimize the error function given in (3). The second part of this section is dedicated to describe the method for passing from a deterministic estimation to a stochastic estimation. This second point of view allows us to get interval estimation of the parameters and some measures related to them.

### Deterministic estimation of the spreading of the COVID-19 epidemic in Spain

We estimate the parameters of model (1) for the first wave of the COVID-19 pandemic in Spain (from February 20 until May 17, 2020). We divide this period into four subperiods according to the restrictions which were imposed by Spanish government at different moments of time: 1) 20/02-12/03; 2) 12/03-1/04; 3) 1/04-21/04; 4) 21/04-17/05. In the first period, when there were no restrictions, the coefficients of the model are assumed to be constant (which gives a good fitting), while in the rest of periods the rate of transmission $\beta \left( t\right)$ and the functions $\gamma _{1}\left( t\right) ,\ \gamma _{2}\left( t\right)$ are piecewise continuous with a finite number of discontinuities and such that in each interval of continuity the form of the functions is the following:

$$\begin{aligned} \beta \left( t\right)= & {} \beta _{0}-\beta _{1}\left( 1-e^{-\alpha \left( t-t_{0}\right) }\right) , \\ \gamma _{i}(t)= & {} \gamma _{0,i}-\gamma _{1,i}\left( 1-e^{-\alpha _{i}\left( t-t_{0}\right) }\right) ,\ i=1,2. \end{aligned}$$

This approach was implemented in [20–22]. We observe also that in the first period, apart of the parameters $\beta ,\ \gamma _{1}$ and $\gamma _{2}$, we estimate the value of the initial condition $E_{0}$ of the variable $E\left( t\right)$.

We take the value of $\sigma$ constant and equal to 1/5 following [23, 24], where the incubation period of the virus was estimated to be five days. Other studies give a larger interval of incubation. (In [25], the value is around 6 days.)

With respect to the parameter $\rho \left( t\right)$, we approximate it by a constant value given by the study of seroprevalence in Spain [26]. According to it, the number of infected people in Spain at the end of May of 2020 was about 2400000 (the 5.2% of the population), whereas at that moment 230000 people were detected by the COVID tests. This implies that during the first wave of the epidemic in Spain the average rate of detection was approximately equal to 0.1.

In order to fit the model, we use the observed values of the variables $D\left( t\right) ,$ $F\left( t\right) ,$ $R\left( t\right)$, that is, the number of detected currently active infected individuals, of detected dead people and of detected recovered people. For the variable $D\left( t\right)$ we use only the number of people who were confirmed to be infected by a PCR test. The observed value for these variables at the moment of time $t_{i}$ will be denoted by $D_{i},\ F_{i}$ and $R_{i}$, respectively. The value $t_{i}$ indicates the current day. Then we fit the model by using as the error function a weighted average of the quadratic error function of each variable, that is,

3 $$\begin{aligned} Error=\alpha _{1}\sqrt{\sum _{i=1}^{n}\left( D_{i}-D\left( t_{i}\right) \right) ^{2}}+\alpha _{2}\sqrt{\sum _{i=1}^{n}\left( F_{i}-F\left( t_{i}\right) \right) ^{2}}+\alpha _{3}\sqrt{\sum _{i=1}^{n}\left( R_{i}-R\left( t_{i}\right) \right) ^{2}}, \nonumber \\ \end{aligned}$$

where $\alpha _{1}+\alpha _{2}+\alpha _{3}=1$. We have chosen $\alpha _{1} =\alpha _{2}=0.35,\ \alpha _{3}=0.3.$ Here, $D\left( t_{i}\right) ,\ F\left( t_{i}\right)$ and $R\left( t_{i}\right)$ stand for the approximative values of these variables at moment $t_{i}$ for a given set of parameters, which are calculated numerically using the standard Runge–Kutta method, whereas *n* is the number of days in the corresponding period. It is worth pointing out that there exist in the literature more accurate semi-numerical methods such as the extended Laplace transform or the multistage Adomian decomposition method (see [27–30]). However, for our purposes the classical numerical methods are enough.

In order to minimize the target function (3), we make use of the heuristic algorithm called differential evolution (see [31, 32]), which is a population-based direct-search algorithm for global optimization. We use the algorithm described in [31]. We denote by *x* a vector containing a population of parameters. First, we must select randomly *N* populations of parameters $X_{0}=\{x_{1,0},\ldots ,x_{N,0}\}$ within suitable bounds. After that, we implement an iterative algorithm by means of the formula

4 $$\begin{aligned} u_{r,i+1}=x_{r,i}+K(x_{r_{3},i}-x_{r,i})+F(x_{r_{1},i}-x_{r_{2},i} ),\ r=1,\ldots ,N, \end{aligned}$$

where $r_{1}\not =r_{2}\not =r_{3}\not =r$ and $F,K\in \left( 0,1\right)$ are selected randomly for each *r*. Then, we select the values of $x_{r,i+1}$ by the rule

$$\begin{aligned} \left\{ \begin{array} [c]{c} x_{r,i+1}=u_{r,i+1}\text { if }u_{r,i+1}\text { is better than }x_{r,i},\\ x_{r,i+1}=x_{r,i}\text { otherwise.} \end{array} \right. \end{aligned}$$

After repeating this process for each $r=1,\ldots ,N$ we obtain the new set of populations:

$$\begin{aligned} X_{i+1}=\{x_{1,i+1},\ldots ,x_{N,i+1}\}. \end{aligned}$$

This process finishes when either we reach a maximum number of iterates or $X_{i+1}=X_{i}$ for some *i*.

The initial values of the variables of our model are given by:

$$\begin{aligned} I_{0}=30\text {, }F_{0}=0,\ R_{0}=0,\ L_{0}=0,\ S_{0}=N-I_{0}-E_{0}-F_{0} -R_{0}-L_{0}. \end{aligned}$$

As we have said before, the value of $E_{0}$ has to be estimated. The value $I_{0}=30$ comes from the fact that the number of detected infected people on February 20 was equal to 3 by using the value $\rho =0.1$, that is, $10\%$ of the infected persons were detected. At that moment, there were neither detected dead people nor detected recovered people (so $F_{0}=0,\ R_{0}=0$), and we extend this to the variable *L*, although this datum is unknown.

We apply the differential evolution algorithm to our problem using a maximum of 1000 iterates and taking *N* equal to 100 in the first subperiod and equal to 200 in the other subperiods. The results in each subperiod are the following:

1. 20/02-12/03:
  $$\begin{aligned} \beta \left( t\right)= & {} \beta _{0}=1.03758,\ E_{0}=162,\\ \gamma _{1}\left( t\right)= & {} \gamma _{0,1}=0.0066337,\ \gamma _{2}\left( t\right) =\gamma _{0,2}=0.014411. \end{aligned}$$
2. 12/03-1/04:
  $$\begin{aligned} \beta \left( t\right)= & {} 0.56457-0.56451(1-e^{-0.084346\left( t-21\right) }), \\ \gamma _{1}\left( t\right)= & {} 0.010016+0.0019473(1-e^{-0.11145\left( t-21\right) }), \\ \gamma _{2}\left( t\right)= & {} 0.0034428+0.082453(1-e^{-0.026258(t-21)}). \end{aligned}$$
3. 1/04-21/04:
  $$\begin{aligned} \beta \left( t\right)= & {} 1.29274\text {-}10^{-16} +0.035546(1-e^{-0.84439(t-41)}), \\ \gamma _{1}\left( t\right)= & {} 0.0091134-0.0038616(1-e^{-0.16832(t-41)}), \\ \gamma _{2}\left( t\right)= & {} 0.05408-0.022434(1-e^{-0.74667(t-41)}). \end{aligned}$$
4. 21/04-17/05:
  $$\begin{aligned} \beta \left( t\right)= & {} 6.33755\text {-}10^{-6} +0.031897(1-e^{-0.045468(t-61)}), \\ \gamma _{1}(t)= & {} 0.0040438-0.0024332(1-e^{-0.047868(t-61)}), \\ \gamma _{2}\left( t\right)= & {} 0.034796-0.0040778(1-e^{-0.032499(t-61)}). \end{aligned}$$

**Fig. 1:**
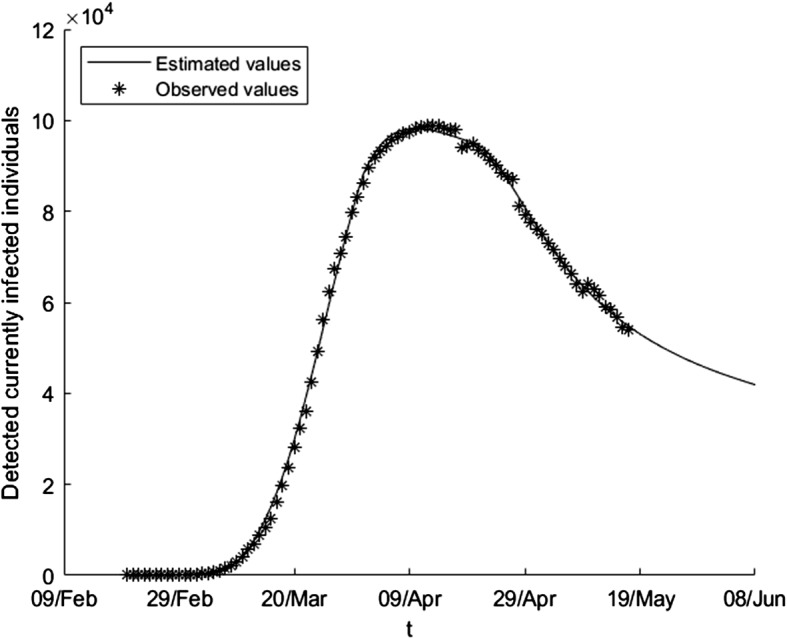
Estimation of detected currently infected individuals using the estimated parameters over the whole period

**Fig. 2:**
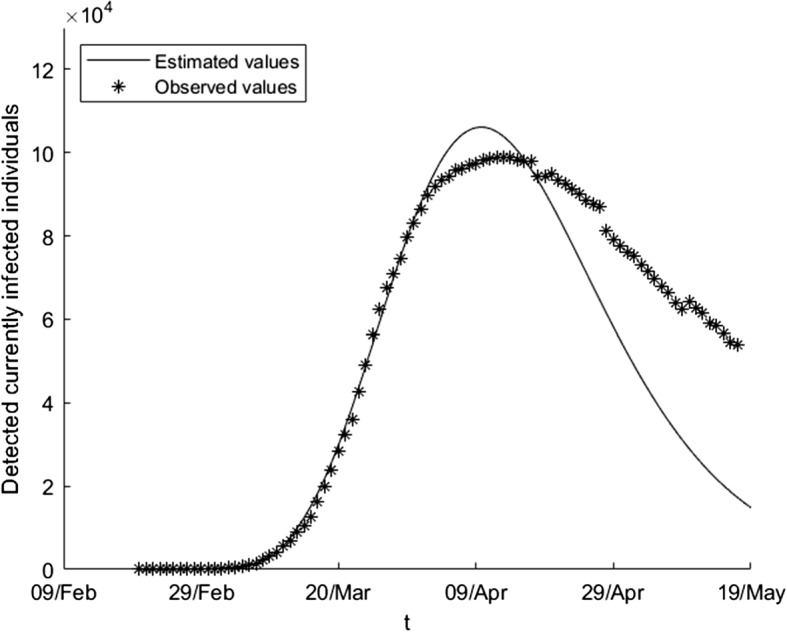
Estimation of detected currently infected individuals using the estimated parameters over the first two subperiods

In Fig. 1, one can see the estimation of the number of currently infected people when we consider the estimated parameter over the whole period, whereas in Fig. 2 we only use the estimated parameters over the first two subperiods (that is, we predict the evolution of the variable using the available information until the first of April). The estimation gives a fairly good picture of the future evolution of the epidemic: it predicts quite good the moment at which the peak of the epidemic is reached, and we come to the correct conclusion that the number of active infected individuals will decay monotonically during May, achieving a low incident rate. Of course, the prediction is not precise in the third and fourth intervals, the reason being that the government restrictions changed twice during this time.

### From point estimation to interval estimation: squeezing the data via bootstrap

In the previous section, we have explained how to manage the problem of estimating the parameters of our model (1) from a deterministic point of view. Thus, the problem is solved via a deterministic paradigm. However, this is not the best paradigm if either the nature of the phenomenon is random or the values of certain magnitudes are subject to uncertainty. In such a case, we consider that it is more appropriate to use a stochastic point of view, that is, to take into account the possibility that either the model or the parameters cannot be correctly specified.

With this purpose, we consider that the model is adequate, but that the principal magnitudes to be estimated are affected by uncertainty. That is, the number of susceptible, infected, dead persons and so on are magnitudes with uncertainty, and then, the value that we observe at a concrete date is one of the probable values that we could observe. At this moment, it is necessary to indicate how we introduce the uncertainty in the method: the way that we propose is to apply bootstrap techniques to the observed data in order to derive the corresponding uncertainty of the magnitude.

#### Obtaining uncertainty from data

In actuarial science, and others, it is common to assume that an stochastic magnitude is unknown, for example the risk of death at age *x*, $q_{x}$. Then, when we observe a value of this variable, $q_{x}^{obs}$, we consider it as a particular realization of the random phenomenon, assuming then that $q_{x}^{obs}=q_{x}+r_{x}$, where $q_{x}$ is the expected value of the variable and $r_{x}$ is a random fluctuation of the magnitude that depends on the probability law of $q_{x}$. In particular, if the variable is the number of dead people at age *x* and it can be modeled via a binomial law, $Bi\left( N_{x},q_{x}\right)$, then each observed value $e_{x}$ can be interpreted as a realization of the phenomenon. Thus, we can obtain an approximation of the probability of death $q_{x}$ by the quotient $\ \hat{q}_{x}=\frac{e_{x}}{N_{x}}$. If the value of $q_{x}$ is known, then we can use Monte Carlo simulations or other similar techniques to obtain multiple scenarios of the variable. When $q_{x}$ is not known, there exist several techniques to estimate the true value of the parameter $$q_{x}\,\ $$using the observed values ${\hat{q}}_{x}$ or $q_{x}^{obs}$. There exist parametric (mortality laws, fit of a family of functions) and nonparametric techniques (mobile average, kernel graduation, wavelet graduation) (see [33–36]). In this work, we consider the nonparametric techniques based on the wavelet graduation introduced in the actuarial field in [10, 37–39], which was used in a satisfactory way in two dimensions in [40]. This technique is based on the wavelet decomposition of a numerical series.

The wavelet decomposition (see [41, 42]) of a numerical series splits the series into two parts: the scale part and the wavelet part. The first part captures the tendency of the series; the second part captures certain details of the series and the random fluctuations. Associated with the *wavelet transform*, there exists the *inverse wavelet transform. *These two transforms are inverse functions in the sense that the inverse wavelet transform applied to the wavelet transform of a series provides us the original values. Wavelet and inverse wavelet transforms enable us to treat the random fluctuations. It is convenient to note that the wavelet decomposition can be done as a* “multiresolution scheme”* [43]. It consists in applying the decomposition sequentially. That is, we say that the level of the decomposition is two if we apply two times the wavelet decomposition, the second time over the scale part of the first decomposition; then, we have two scale parts and two wavelet parts. Repeating this procedure, we have a multiresolution scheme of order *n*.

The wavelet graduation treats to isolate the random fluctuations and to estimate the expected (or real) values; with this aim, we use the standard technique named *thresholding*. This technique is based on treating the wavelet part (it is known that this part contains the random fluctuations if they are Gaussian [42]); the treatment of the wavelet part can be done using hard or soft thresholding, among other techniques. In this work, we consider hard thresholding, that is, the values which are less than a certain quantity (the threshold) are transformed to 0. The value of the threshold considered in this work is proportional to the expression $2\sigma \sqrt{2\log N}$ ([42, 44, 45]), where $\sigma$ is equal to the standard deviation of the wavelet part of the first level, and *N* is equal to the number of values which are considered. This method is given in detail in some previous papers (see [10, 37–39]). From here, it is straightforward to obtain the graduated values (as an approximation of the expected values) using the inverse wavelet transform on the initial scaling part jointly with the modified (thresholding) wavelet part. Then, the differences between the initial (observed) values and the graduated values estimate the random fluctuations.

**Fig. 3:**
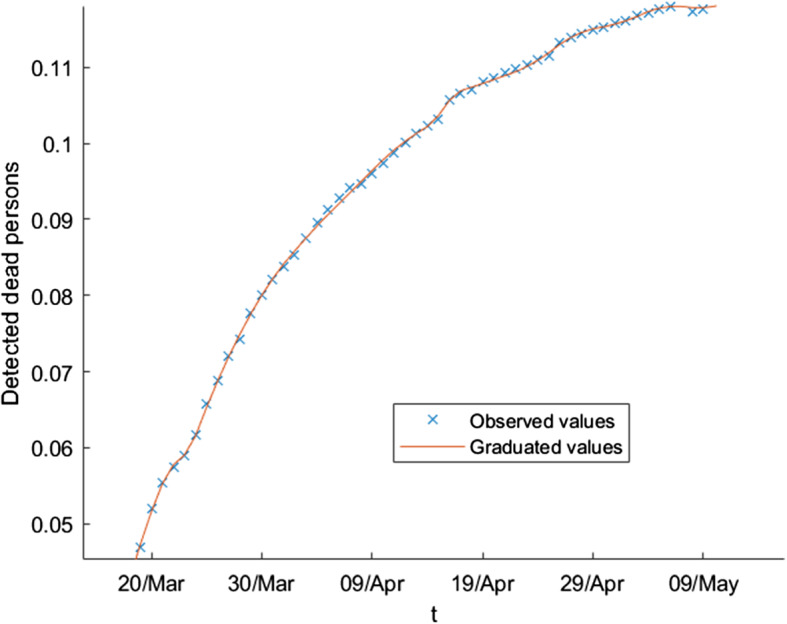
Crude rates of the number of detected dead people

**Fig. 4:**
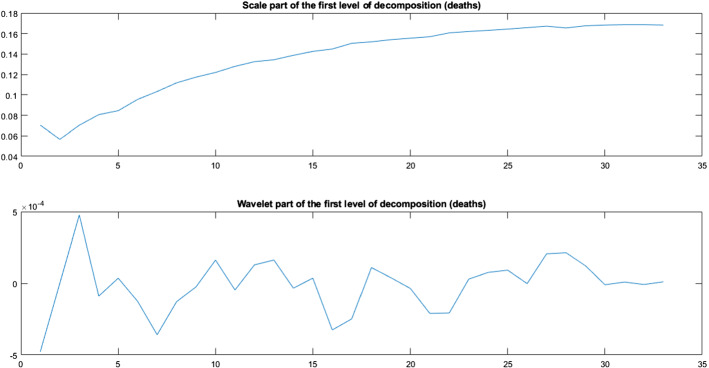
Wavelet decomposition (crude rates of deaths)

The wavelet graduation process produces two series of values: the expected values and the random fluctuations. Let us apply this technique to the variable *F* (the number of detected dead individuals) in our model (1) for the evolution of the epidemic in Spain in the period from March 10, 2020, until May 17, 2020. Instead of using *F* directly, we consider the crude rate of this variable $q_{t}$ (the probability of death at the moment of time *t*), defined by the quotient

$$\begin{aligned} q_{t}=\frac{F_{t}}{T_{t}}, \end{aligned}$$

where $F_{t},\ T_{t}$ are, respectively, the number of detected dead people and the total number of detected infected people by a PCR test at the moment *t*. Figure 3 shows the observed values of the variable $q_{t}$ and its graduated values in the period from March 10, 2020, until May 17, 2020; Fig. 4 shows, for the first level of the decomposition, the scale part (top part of the panel) and the random fluctuation extracted from the observed values (down part of the panel).

Now, we can stop and give an estimate (a point estimation) via the expected values (wavelet graduated values) or we can go further and use the random fluctuations, which were calculated previously in the wavelet graduation, to obtain other possible initial values, named *synthetical observed values*. Among the techniques that we can use to do this, the bootstrap technique (see [1, 2]) emerges as an appropriate technique. This technique is based on random sampling (with replacement), and it is a special case of the resampling techniques. The idea of the bootstrap technique is that the inference that could be done over the whole population is approximated by the inference that is done over a sample. In this sense, we do not know the population of the variable of interest, but only the observed values. However, via wavelet graduation, we know an approximation of the expected values and the random fluctuations. This last part, i.e., the random fluctuations, is the object of the bootstrap technique with the aim to obtain other synthetical initial values. The random sampling (with replacement or not) technique requires that the values to be resampled form a s.r.s. (simple random sample) from the same probability distribution. If this condition is true, then we can apply the bootstrap method *B* times to obtain *B* samples. This procedure was introduced in [10], where it was applied to the actuarial field.

In our model (1), we will use three variables: $E_{t}$ (the number of exposed individuals at moment *t*), $D_{t}$ (the number of detected currently infected persons at moment *t*) and $F_{t}$ (the number of detected dead persons at moment *t*). Then, from the observed values we can derive bootstrap series (*B*-series for short, also called *B*-realizations) of each variable *D* and *F*, denoted by: $\left\{ D_{1}^{b},D_{2}^{b},\ldots ,D_{t} ^{b}\right\} _{b=1}^{B}$; $\left\{ F_{1}^{b},F_{2}^{b},\ldots ,F_{t} ^{b}\right\} _{b=1}^{B}$. It is important to point out that we do not have observed values for the variable *E*, so we use its estimated values after solving numerically system (1). Moreover, they are constant over all realizations, and we use them to obtain the bootstrap series of the variables *D* and *F*.

##### Remark 1

The variables involved in this study are not independent. For example, we have calculated that there exists a linear correlation between the variables *D* and *F*. The bootstrap series and the estimated values of these variables show a similar level of linear correlation. In a sense, this confirms that the method to generate different scenarios is adequate.

We consider an improvement in this work that we have used resampling techniques of numerical simulation in a system of differential equations, which allows us to move from a deterministic framework to a stochastic one, taking into account in this way uncertainty. Techniques based on the Cholesky decomposition or similar ones can be considered in a future work.

##### Remark 2

As we will see later, we will solve model (1) *B* times, one for each bootstrap series of input data.

#### Steps and technical details of the numerical implementation

*Series of values to be considered*

It is not convenient to apply the above procedure directly to the observed values of the variables because they have exponential growth. Thus, as the magnitude of the involved variables are very different at each time, it is difficult to apply the method. To avoid this problem, we apply the method on the following ratios: $\frac{D_{t}}{E_{t}}$ and $\frac{F_{t}}{D_{t}}$. We note that this transformation is reversible and allows us to recover the initial values or, if it is of our interest, other related values with the same order of magnitude (resampled values).

*Treatment of the initial series*

**Fig. 5:**
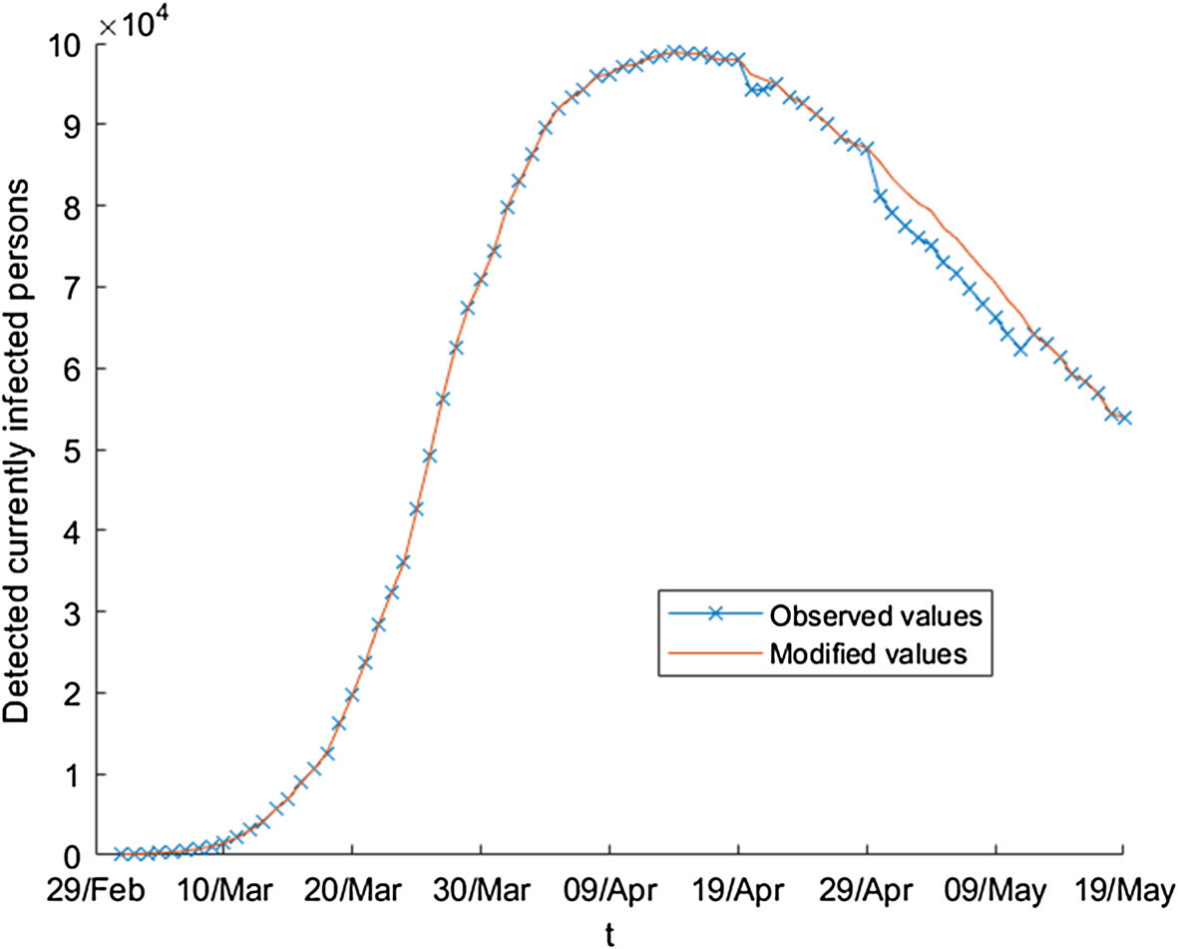
Modified values of the absolute number of detected currently infected persons

**Fig. 6:**
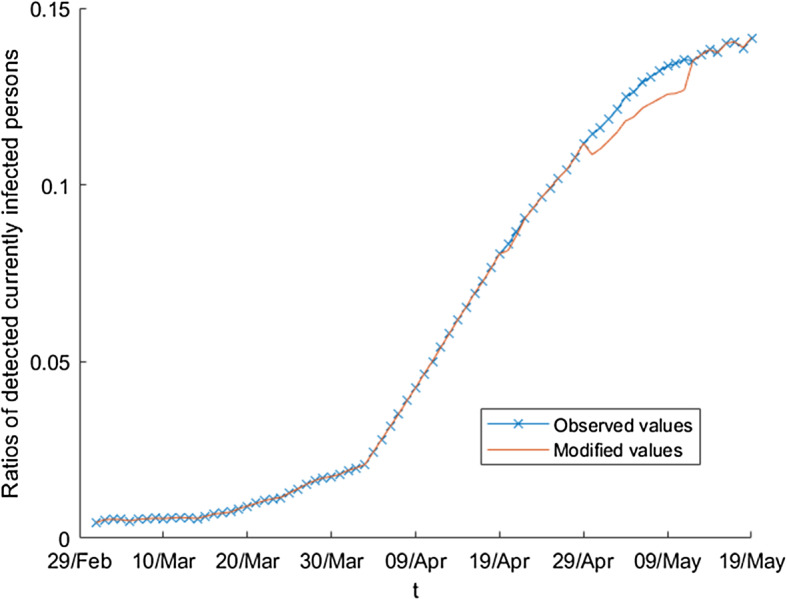
Modifed values of the ratios of detected currently infected persons

When the observed values are not correct, the inference based on them are incorrect as well, and then, it is evident that the implications can be very serious. In this situation, it is necessary to treat the observed values in order to reduce the errors that could exist. Analyzing the observed values, it is possible to detect the existence of some errors or inconsistencies. In the initial pandemic period (the *first wave*), data were subject to mistakes of different kind coming, for example, from incorrect registrations, duplicity or delays in the communication, with the corresponding rectifications and corrections, as we can see in [46, 47] or [48]. Then, the treatment of the initial series is necessary, but not only in relation to the corrections of the Spanish government but also to technical (and reasonable) aspects such as the continuity of the series or the magnitude of the values in adjacent times, which should be similar. Figures 5 and 6 show for the variable $D_{t}$ and the ratios $\frac{D_{t}}{E_{t}},$ respectively, the original values and the corrections to them.

The treatment of the observed values can be summarized as:

1. We consider the observed values of infected people and determine, empirically, that in the days 59–61 (April 18–20) they have a small error. Also, the values in the period between the days 69–80 (from April 28 to May 9) have a different level than in the previous part of the curve.
2. We implement the next corrections:
  - We modify the values at days 59 and 60 as: $I_{59} ^{{\text {mod}}}=\frac{I_{58}+I_{60}}{2}$, $I_{60}^{{\text {mod}} }=\frac{I_{59}^{{\text {mod}}}+I_{61}}{2}$.
  - For the period from April 28 to May 9, we consider the next levels: $s_{1}=I_{69}-I_{68};$ $s_{2}=I_{70}-I_{69};$ $s_{3}=I_{71}-I_{70};$ $s_{4}=I_{72}-I_{71}$. In addition, we define $s=s_{1}-\frac{s_{2}+s_{3} +s_{4}}{3}$. Then, $I_{k}^{{\text {mod}}}=I_{k}-s$, $k=69,\ldots ,80$.

We note that the previous transformations are necessary to recover, later, the values of the variable in the observed magnitude.

##### Remark 3

We note that $E_{t}$ is a subjacent variable, and it is not possible to know its true values. Then, as told before, we use its estimated values by the deterministic method through system (1). These values are the same for all synthetic scenarios.

*Details of the wavelet graduation*

Subsequently to the treatment of the observed values, we apply the wavelet graduation on the treated values. The details of this procedure can be found in [2, 10, 37, 39, 41–45]. We use a multiresolution squeme with three levels jointly with the wavelet family *biorthonormal 3.3* (see [41, 49, 50]). To split the observed values into the expectation and random fluctuations, we use the thresholding technique with automatic threshold proportional to the value estimated by $2\sigma \sqrt{2\log N}$ (see [44]), where *N* is equal to the number of observational days and $\sigma$ is the standard deviation of the wavelet part from the first level of multirresolution scheme. We obtain that, for the observed series of crude rates of the variables *D* and *F*, the values of the thresholds are closed to $1.1096\times 10^{-3}$ and $1.0521\times 10^{-3}$, respectively.

*Treatment of the heteroscedasticity: Pearson residuals*

**Fig. 7:**
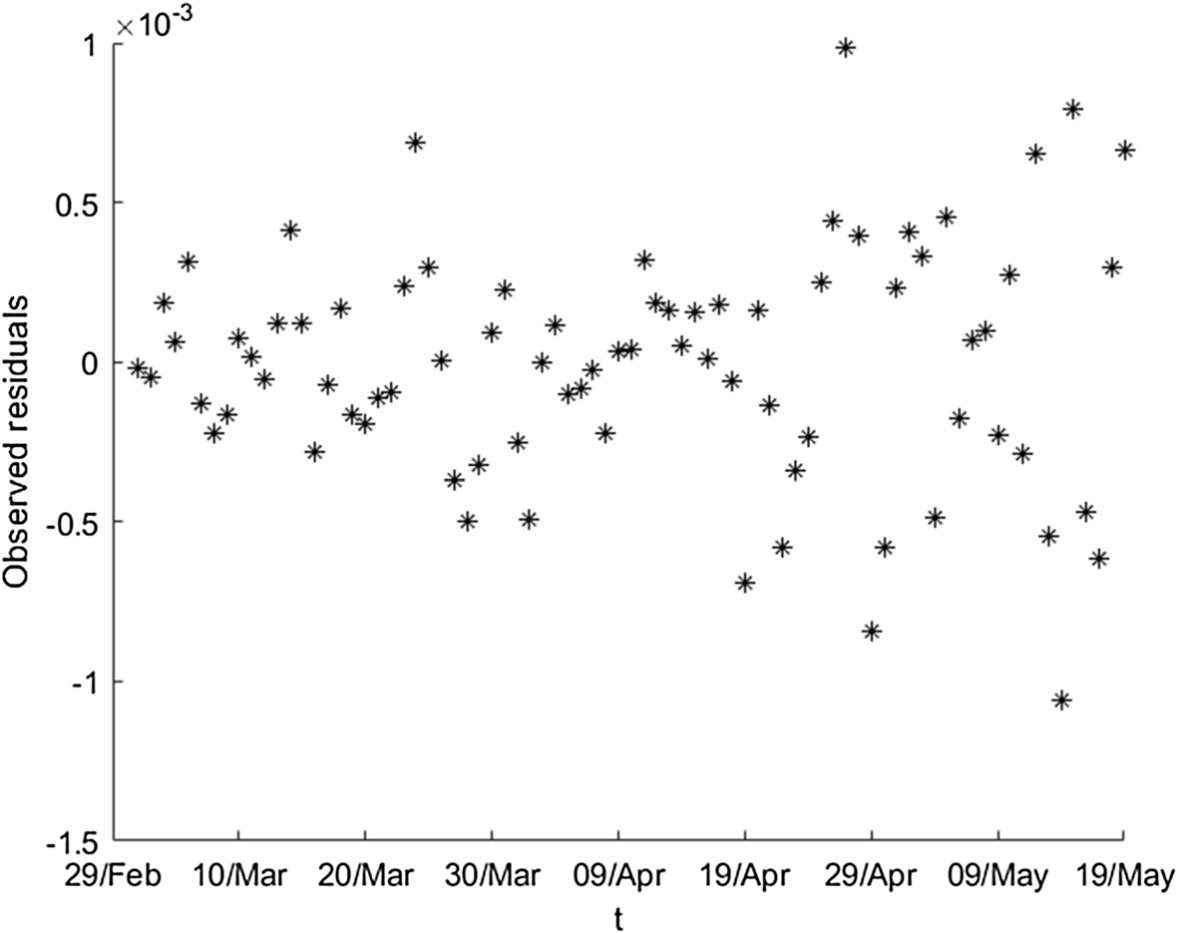
Residuals of the wavelet graduation for the ratios of detected currently infected persons

In Fig. 7, we can observe that the residuals are not homoscedastic (i.e., they do not have the same variance), which indicates us that the bootstrap technique cannot be applied directly, because all of the residuals are not equally distributed, and then, we cannot consider them as a simple random sample.

To avoid the problem of the heteroscedasticity, which is necessary in order to apply the resampling techniques, we make use of a transformation of the residuals in such a way that the transformed data can be considered as a random simple sample of the same law. In particular, we apply the *Pearson transformation* [51], for which the new values are normally distributed. Also, we consider that this transformation is appropriate because it has a explicit reverse transformation.

To visualize how we apply this transformation, we will use a particular series of observed values; for example, the number of detected currently infected individuals $\left\{ D_{t}\right\}$ and the number of exposed individuals $\left\{ E_{t}\right\}$. Then, we follow the next sequence of steps, where the last one refers to the Pearson transformation:

1. We estimate the ratios $q_{t}=\frac{D_{t}}{E_{t}},\ t=1,\ldots$
2. We treat the inconsistencies of the series and we obtain a first estimation of the real values of $q_{t}$, denoting them by $\left\{ \hat{q}_{t}\right\}$.
3. We apply the wavelet graduation on $\left\{ {\hat{q}}_{t}\right\}$ obtaining two series of values: the wavelet graduated values $\left\{ \overset{\circ }{q}_{t}\right\}$ (which approximate the expected values by the scale part), and the random fluctuations $\left\{ r_{t}\right\}$ (from the wavelet part).
4. It is evident from Fig. 7 that the residuals are not similar in magnitude. We are interested in a transformation such that the new values are Gaussian or very closed to this probability distribution. The expression
  5 $$\begin{aligned} r_{t}^{Pearson}=\frac{E_{t}q_{t}-E_{t}\overset{\circ }{q}_{t}}{\sqrt{E_{t}\overset{\circ }{q}_{t}\left( 1-\overset{\circ }{q}_{t}\right) }} \end{aligned}$$
  describes the Pearson transformation [51] and provides us a new series of residuals, $\left\{ r_{t}^{Pearson}\right\} ,$ verifying that the values are Gaussian or close to such distribution.

**Fig. 8:**
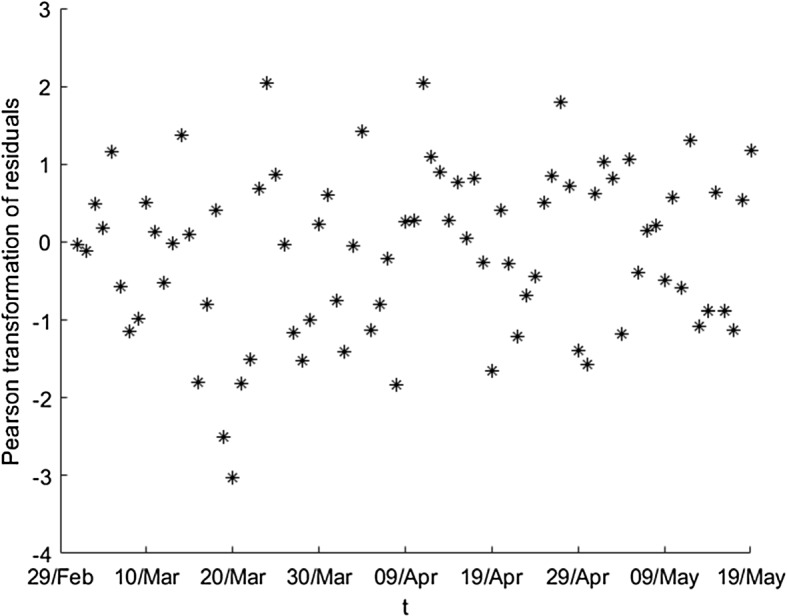
Pearson transformation of residuals for the ratios of detected currently infected persons (adimensional number)

Figure 8 shows the (new) Pearson residuals. Visually we can observe that all of them are of the same magnitude. Moreover, applying the Jarque–Bera test (see [52, 53]) we obtain that the *p* value is closed to 0.459; then, we accept the hypothesis of normality of the Pearson residuals.

*Resampling method and resampled series*

**Fig. 9:**
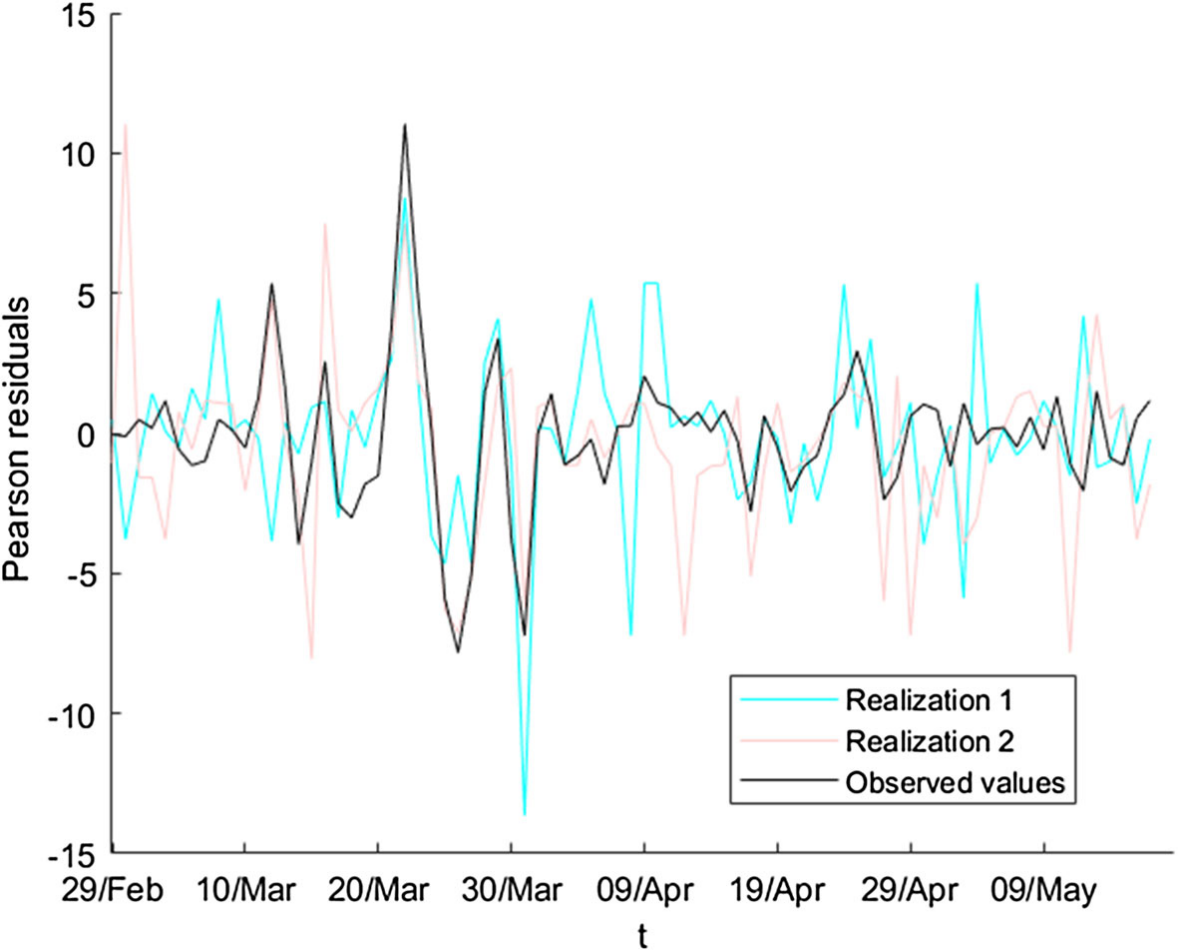
Pearson residuals of detected currently infected persons (adimensional number)

In order to generate equally probable synthetic scenarios, we must assume that the residuals $\left\{ r_{t}^{Pearson}\right\}$ form a simple random sample. In this situation, we can apply the bootstrap technique on them, and combining this resampling method with the expected values, we obtain the bootstrap scenarios. To do this, we apply resampling with replacement over $\left\{ r_{t}^{Pearson}\right\}$, and then, we obtain $\left\{ r_{t}^{P,1}\right\} _{t}$. If we repeat this procedure, we can obtain *B* resampled residuals, denoted by $\left\{ \left\{ r_{t}^{P,b}\right\} _{t}\right\} _{b=1}^{B}$. Figure 9 shows several bootstrap residuals.

We recall that the bootstrap residuals are Pearson residuals, so we need to recover the original values. To do this, we use the inverse of the Pearson transformation (5). Namely, for each *b* and *t*, we calculate the bootstrap value $q_{t}^{b}$, using the formula

$$\begin{aligned} q_{t}^{b}=\overset{\circ }{q_{t}}+\frac{r_{t}^{P,b}}{E_{t}}\sqrt{E_{t} \overset{\circ }{q}_{t}\left( 1-\overset{\circ }{q}_{t}\right) }, \end{aligned}$$

where $\overset{\circ }{q_{t}}$ is equal to the expected value via the wavelet graduation; $E_{t}$ is the number of exposed individuals; and $r_{t}^{P,b}$ is the bootstrap residual obtained in the previous step (all of them at moment *t*).

Finally, in order to obtain the bootstrap values of the detected currently infected persons $\left\{ D_{t}^{b}\right\} _{b=1}^{B}$, we use that $q_{t}=\frac{D_{t}}{E_{t}}$ and replace $D_{t}$ by $D_{t}^{b}$; then, we obtain the values via the expression

$$\begin{aligned} D_{t}^{b}=q_{t}^{b}E_{t}\text {, }b=1,\ldots ,B;\text { } t=1,\ldots ,T\text {.} \end{aligned}$$

We note that it is necessary to know the values of $\left\{ E_{t}\right\}$. This series is fixed in all the *B* samples, and its values are estimated initially using the SEIR model (1). Also, we observe that we can obtain a point estimation via wavelet graduation, using the expression

6 $$\begin{aligned} \overset{\circ }{D_{t}}=\overset{\circ }{q_{t}}E_{t}\text {, }t=1,\ldots ,T\text {.} \end{aligned}$$

**Fig. 10:**
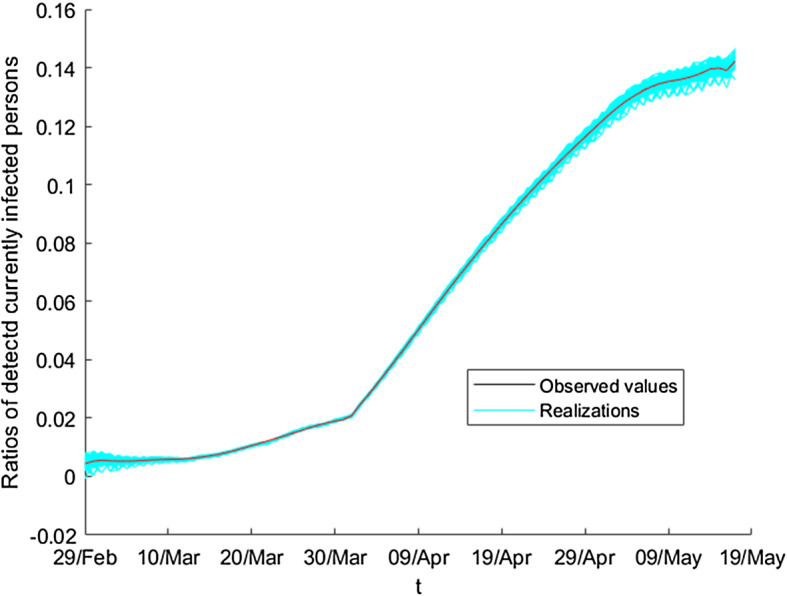
Ensemble of bootstrap series of the ratios of detected currently infected persons

**Fig. 11:**
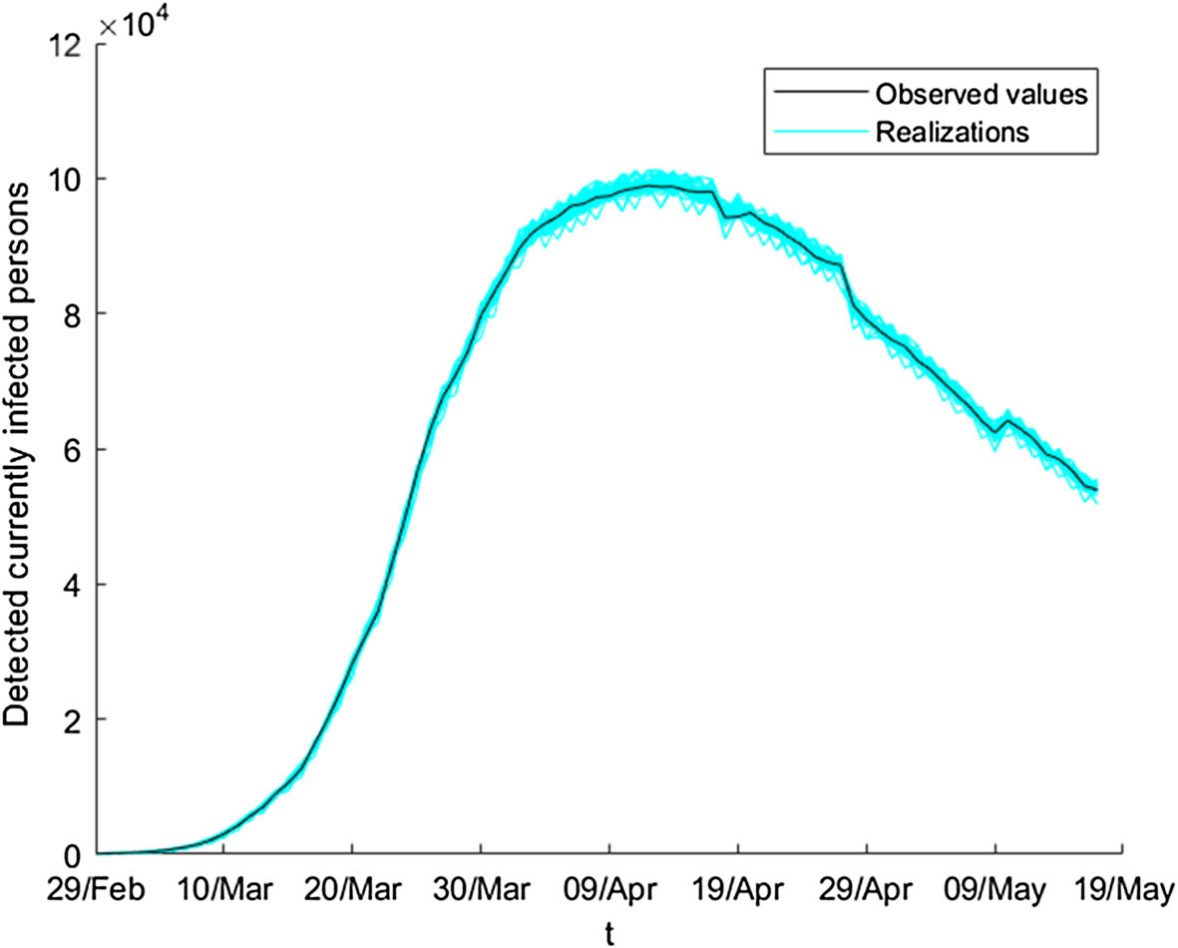
Ensemble of bootstrap series of the absolute value of detected currently infected persons

Figures 10 and 11 show some bootstrap series of the variable $D_{t}$ jointly with the observed values, for the ratios $\frac{D_{t}}{E_{t}}$ and for the same variable, respectively.

**Fig. 12:**
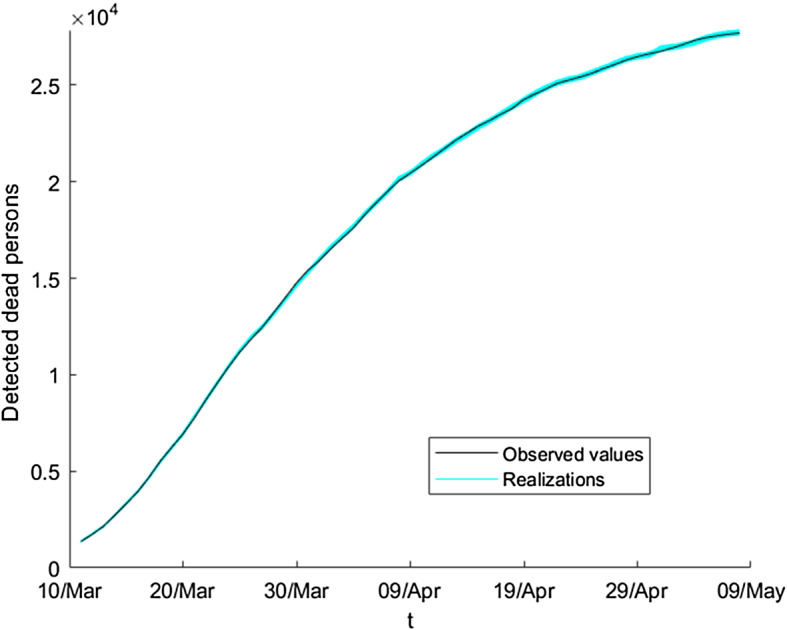
Ensemble of bootstrap series of the absolute value of detected dead persons

Similarly, we obtain the graduated and the resampled values of the number of detected dead people, $\overset{\circ }{F_{t}}$ and ${F_{t}^{b},\ b=1,\ldots ,B}$. Figure 12 shows some bootstrap series of this variable jointly with the observed values.

*Applying the SEIR model: bootstrap estimates of the spreading of the COVID-19 epidemic in Spain*

Once we have the bootstrap series of the variables *D* and *F*, for each $b\in \left\{ 1,\ldots ,B\right\}$ we use the values $\{D_{t}^{b} \},\ \{F_{t}^{b}\}$ and apply the method of estimation of the parameters of model (1) given in Sect. 3.1. The sample of observed values for the variable *R* of detected recovered individuals is the initial one and remains constant for any *b*. This procedure provides us *B* estimates of the parameters of model (1), jointly with the estimation of the variables of interest for each *b*, denoted by $\left\{ {\widetilde{S}}_{t} ^{b}\right\} ,\ \left\{ {\widetilde{E}}_{t}^{b}\right\}$,$\ \left\{ {\widetilde{D}}_{t}^{b}\right\}$, $\left\{ {\widetilde{F}}_{t}^{b}\right\}$, etc.

**Fig. 13:**
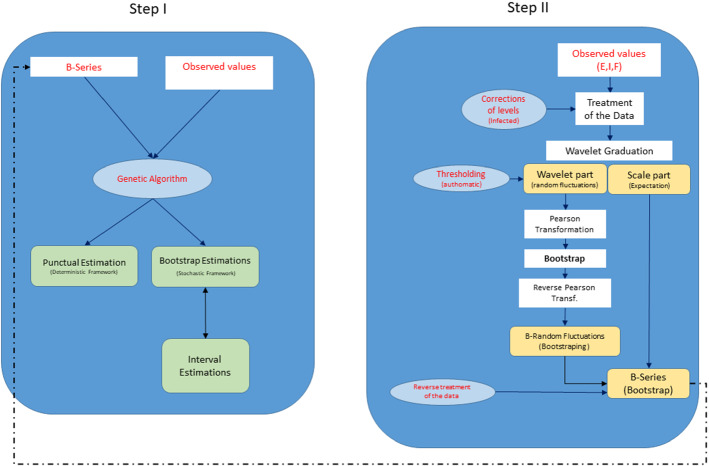
Diagram of the procedure

Figure 13 shows a mental framework of the procedure.

#### Using uncertainty

The procedure starts with the estimates of the parameters of model (1) for each *b*. Then, we can forecast the values of the variables of interest and obtain the predicted values $\left\{ {\widetilde{D}}_{t} ^{b},{\widetilde{F}}_{t}^{b}\right\} _{t=1}^{T_{H}},$ for each *t* until a time horizon denoted by $T_{H}$. Of course, we obtain also predictions for the other variables of the model (*S*, *E*, *R* etc.), but we will focus on the variables *D* and *F*. This allows us to define several types of forecast values: point estimation and interval estimation. Namely:

- Several point estimations derived from the bootstrap are:
  - The graduated values of the wavelet graduation, the tendency part.
  - Using the *B* samples, we calculate, for each time, the mean estimation.
  - Similarly to the mean, we calculate, for each time, the median estimation.
- The interval estimation allows us to estimate the rank variation and the quantile approximation:
  - Using all the bootstrap estimations, we can construct an interval estimation for each $t\in \left[ 1,T_{H}\right]$:
    $$\begin{aligned} {\widetilde{F}}_{t}^{b}\in \left[ {\widetilde{F}}_{\min ,t},{\widetilde{F}}_{\max ,t}\right] , \end{aligned}$$
    with ${\widetilde{F}}_{\min ,t}=\min \left\{ {\widetilde{F}}_{t}^{b},b=1\ldots B\right\}$ and ${\widetilde{F}}_{\max ,t}=\max \left\{ {\widetilde{F}}_{t} ^{b},b=1\ldots B\right\}$. This interval is called the *B*-*rank interval at time t*, and it is denoted by $Z_{r,F}^{t}.$
  - Also, we can estimate the *B-*$$\alpha $$-*quantile interval* *at time *$$t $$ of level $$\alpha \in \left[ 0,1\right] $$, denoted by $$Z_{\alpha ,F}^{t}$$:
    $$\begin{aligned} Z_{\alpha ,F}^{t}=\left[ {\widetilde{F}}_{\alpha ,t},{\widetilde{F}}_{\left( 1-\alpha \right) ,t}\right] , \end{aligned}$$
    with ${\widetilde{F}}_{\alpha ,t}$ equal to the $\alpha -$quantile of the sample $\left\{ {\widetilde{F}}_{t}^{b},b=1\ldots B\right\}$.

We note that the definitions of rank interval and quantile interval are the same for ${\widetilde{E}}_{t}^{b}$ and ${\widetilde{D}}_{t}^{b}$, obtaining $Z_{r,E}^{t}$, $\ Z_{\alpha ,E}^{t}$ and $Z_{r,D}^{t}$, $\ Z_{\alpha ,D}^{t}$, respectively. The other variables of the model can be treated in a similar way.

#### Measuring uncertainty

Further, in order to measure the usefulness of introducing estimation via bootstrap intervals, we will consider the following indicators:

1. For each variable *D* and *F*, we calculate the percentage of observed values lying in the corresponding *B*-rank interval:
  $$\begin{aligned} P_{r,F}=\frac{\sum _{t=1}^{T}\chi _{Z_{r,F}^{t}}\left( F_{t}\right) { \ }}{T}, \end{aligned}$$
  with $\chi _{Z_{r,F}^{t}}\left( F_{t}\right)$ denoting the characteristic function, that is,
  $$\begin{aligned} \chi _{Z_{r,F}^{t}}\left( F_{t}\right) =\left\{ \begin{array}[c]{l} 1\quad \text { if }F_{t}\in Z_{r,F}^{t},\\ 0\quad \text { otherwise.} \end{array} \right. \end{aligned}$$
  Similarly, we define the indicator that measures the percentage of observed data lying within the $\alpha -$quantile interval:
  $$\begin{aligned} P_{\alpha ,F}=\frac{\sum _{t=1}^{T}\chi _{Z_{\alpha ,F}^{t}}\left( F_{t}\right) { \, }}{T}.\ \end{aligned}$$
2. Another way to measure the suitability of the bootstrap interval estimation is in terms of error. It is possible to measure the error that each point estimation produces in absolute and in relative terms:
  $$\begin{aligned} MAE&=\frac{1}{T}\sum _{t=1}^{T}\left| \overset{\circ }{X_{t}}-X_{t} ^{obs}\right| ,\\ MRE&=\frac{1}{T}\sum _{t=1}^{T}\frac{\left| \overset{\circ }{X_{t}} -X_{t}^{obs}\right| }{X_{t}^{obs}},\\ MRqE&=\frac{1}{T}\sum _{t=1}^{T}\frac{\left( \overset{\circ }{X_{t}} -X_{t}^{obs}\right) ^{2}}{X_{t}^{obs}}, \end{aligned}$$
  where $\overset{\circ }{X_{t}}$ refers to the point estimation (deterministic estimation, wavelet graduation, bootstrap mean or bootstrap median) of the variable of interest (*D* or *F*) and $X_{t}^{obs}$ stands for the observed value of the same variable. Here, MAE, MRE and MRqE stand for the mean absolute error, the mean relative error and the mean relative square error, respectively.

#### Sources of information and software

The data are taken from the Spanish Health Ministery [54] and DATADISTA [55].

The treatment of the data, calculus and estimation has been made using MATLAB R2020a Update (9.8.0.1417392) 64 bits.

## Bootstrap estimation

In this section, we will apply the techniques described in the previous section in order to estimate the values of the variables of interest using the proposed SEIR model over all bootstrap series of detected currently infected and detected dead individuals.

**Fig. 14:**
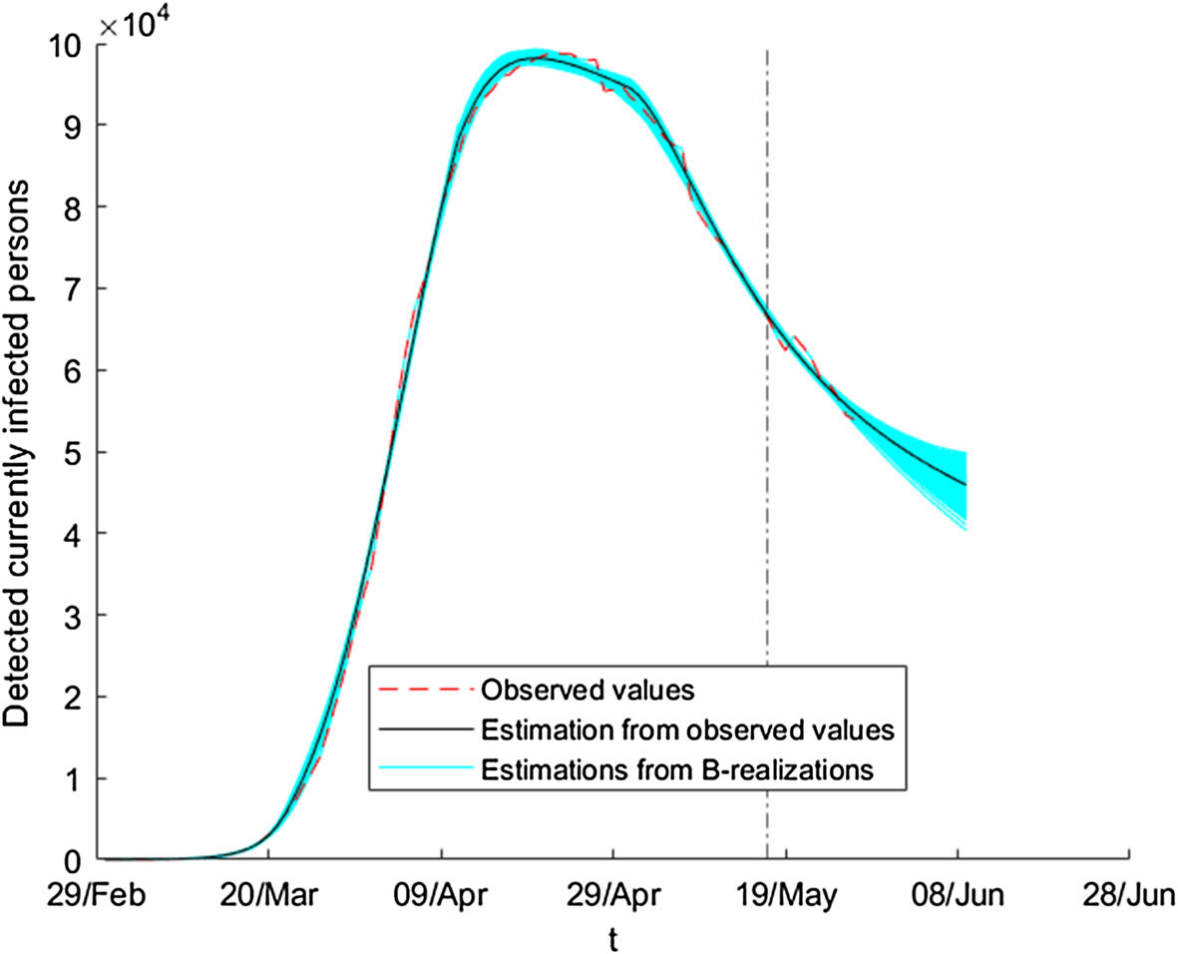
Ensemble of estimated absolute values of detected currently infected persons

**Fig. 15:**
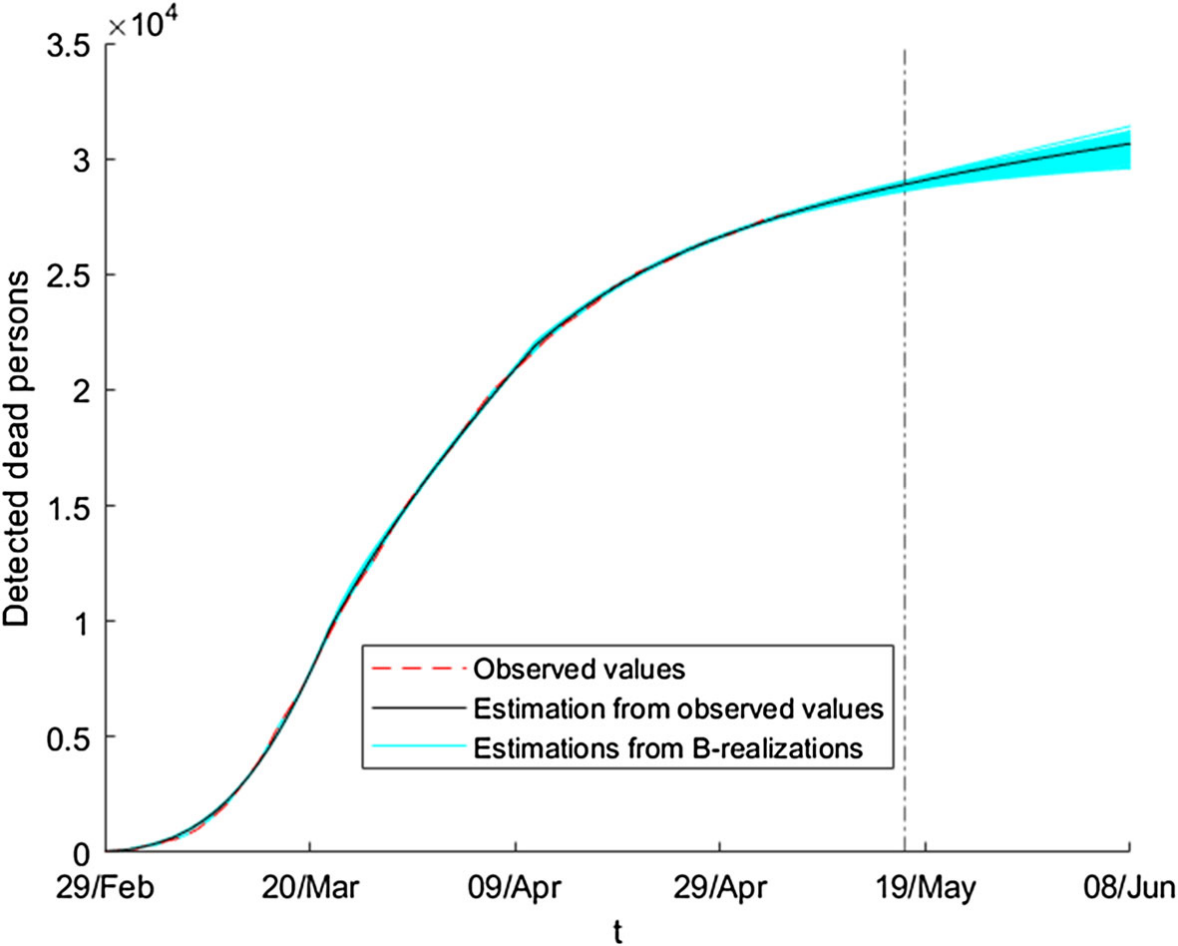
Ensemble of estimated absolute values of detected dead persons

Figures 14 and 15 show, for these two variables, the initial estimation obtained from model (1) for the observed data jointly with the estimations given by using bootstrap series as input data instead of the observed data. The dashed vertical line splits the figure into two regions (periods): the observation period (left-hand side) and the forecasting period (right-hand side).

We observe in Figs. 14 and 15 that all values of the initial estimation are between the corresponding minimum and maximum values of the ensemble of estimations using the bootstrap series. Using this property we can construct, for each time (day), the corresponding bootstrap confidence interval of the initial estimation. The construction of these intervals, for the level of significance $\alpha$, is made using the next expression:

7 $$\begin{aligned} IC_{t,\alpha }= & {} \left[ \theta _{t,1}^{x},\theta _{t,2}^{x}\right] ,\nonumber \\ \theta _{t,1}^{x}= & {} {\overline{x}}_{t}-z_{\frac{\alpha }{2}}{\widetilde{\sigma }} _{t,x}\text {, }\theta _{t,2}^{x}={\overline{x}}_{t}+z_{\frac{\alpha }{2} }{\widetilde{\sigma }}_{t,x}, \end{aligned}$$

where *x* represents the variable of interest, *D* or *F*; for each *t*, ${\overline{x}}_{t}$ is the mean of all estimates and ${\widetilde{\sigma }}_{t,x}$ is the standard deviation. $z_{\frac{\alpha }{2}}$ denotes the value such that $p(Z\ge z_{\frac{\alpha }{2}})\le \frac{\alpha }{2}$, where *Z* is a normal distribution function with expectation 0 and variance 1. For example, if $x=D$, then these quantities are calculated by the standard formulas:

$$\begin{aligned} {\overline{D}}_{t}&=\frac{1}{B}\sum _{b=1}^{B}{\widetilde{D}}_{t}^{b},\\ {\widetilde{\sigma }}_{t,D}^{2}&=\frac{1}{B}\sum _{b=1}^{B}\left( {\widetilde{D}}_{t}^{b}-{\overline{D}}_{t}\right) ^{2}. \end{aligned}$$

Using this construction, we can check whether the initial estimations ${\widetilde{D}}_{t}$ or ${\widetilde{F}}_{t}$ lie in the corresponding interval $IC_{t,\alpha }$. We note that the proportion of times satisfying this property depends on the level of $\alpha$. In this sense, we have found that, up to $\alpha =0.2713$ (for detected infected persons) and $\alpha =0.1835$ (for dead persons), the initial estimations lie in $IC_{t,\alpha }$ for all days in the period from February 20 until May 17. We estimate the intervals $IC_{t,\alpha }$ and their corresponding size of uncertainty. In this sense, we estimate the relative size, with respect to the observed values, of the confidence interval for each day:

$$\begin{aligned} \frac{\theta _{t,2}^{D}-\theta _{t,1}^{D}}{D_{t}^{obs}}. \end{aligned}$$

Using this expression, we find that, for $\alpha =0.27143$, on March 1 the relative size is less than $10\%$; this value decreases quickly to $2\%$ on March 10, taking values closed to $0.4\%$ on March 22 and later.

In order to highlight the usefulness of the proposed method, we could also calculate the percentage of observed data lying either within the minimum and maximum values of the ensemble of estimations using the bootstrap series or in the confidence interval (7) with $\alpha =0.99$. In both cases, the value is closed to $60\%$ for the variables *D* and *F*. We note that these values are big enough. They are not indicators of goodness of the method; they must be interpreted as qualitative indicators of proximity between the observed data and the ensemble of estimations.

**Fig. 16:**
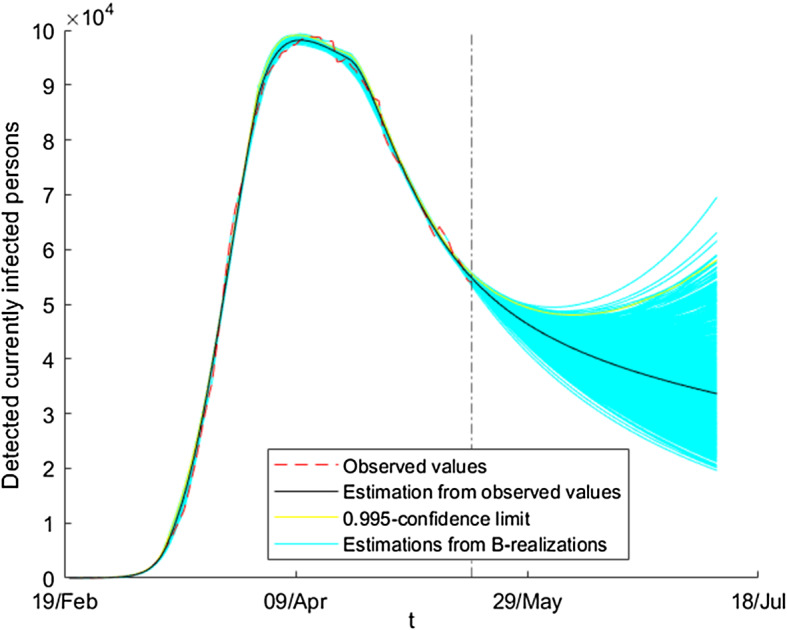
Ensemble of estimated absolute values of detected currently infected persons

In order to evaluate the goodness of the method, we consider more appropriate to compare the series of initial estimated values versus the set of bootstrap estimations.

Now, we will calculate the MRE indicator for the series of detected currently infected persons using the central series: a) the initial estimations; b) the graduated values via wavelet; c) the mean over all bootstrap estimated series. We obtain that the values are 0.0610, 0.0489 and 0.0538, respectively. In the case of the detected dead individuals, the values are 0.05695, 0.03837, and 0.03948, respectively. The first conclusion we come to is that the estimations that are carried out with the graduated values or with the mean of the ensemble of bootstrap estimations have less error than the estimations which use uniquely the observed values.

Also, analyzing the series of errors for detected dead persons, we observe that the percentage of times when one of these series is better than the others are $28\%$, $43\%$ and $29\%$, respectively. That is, in $43\%$ of the days, the estimations made with the wavelet graduated values have less error than the estimations made with the initial estimation or with the mean of the bootstrap estimations. The same interpretation is made for the initial estimation, which has a better estimation in $28\%$ of the days; finally, the mean of the ensemble of the bootstrap estimations is better than the other two in $29\%$ of the days. For detected currently infected persons, these percentages are $39\%$, $42\%$ and $19\%$, respectively.

Finally, for each of these three central series we compute the maximum relative error and the mean of the relative errors when the best approximation is one of the other two:

1. For detected dead people: 8
2. For detected currently infected people: 9

We deduce that when the best estimation is obtained, but one of the other two approximations, the worst value for both the maximum error and the mean of the error, corresponds to the initial estimation. This allows us to assure that, in the described sense, the approximations done using the wavelet graduation and the bootstrap technique are more consistent or robust.

The second conclusion we come to is that the proposed procedure allows us to manage the uncertainty of the data in a more appropriate way than using the punctual estimation by taking into account only observed data.

With respect to the prediction of the evolution of the epidemic after May 17, although the initial estimation predicts a constant decayment of the number of infected people, this is not the case for all the bootstrap estimations. In fact, as can be seen in Fig. 16, in part of them there is a change in trend leading to a new wave of the pandemic. This reflects the fact that small perturbations can change dramatically the situation. In fact, it is a well-known fact in actuarial science that modeling of adverse disasters is complex. In this sense, Monte Carlo’s simulations are useful for the prediction of such phenomena. On the other hand, as indicated by the International Actuarial Association in [56], in order to forecast extreme situations such as the 1918 influenza pandemic or the Black Friday, it is convenient to use the significant level $\alpha =0.005$ in the estimations, so that extreme phenomena occur only one in 200. In this sense, the method of this work allows us to obtain several scenarios so that after calculating the corresponding quantile the decision maker can make appropriate decisions. In our case, a new wave starts in 27% of the series, which is a very high probability from the actuarial point of view. Hence, the conclusion is that there is a big risk of an adverse disaster and, in consequence, some measures should be implemented. In fact, as we know, in July of 2020 a new wave of the pandemic started in Spain.
